# The diagnostic accuracy of artificial intelligence in thoracic diseases: A protocol for systematic review and meta-analysis

**DOI:** 10.1097/MD.0000000000019114

**Published:** 2020-02-14

**Authors:** Yi Yang, Gang Jin, Yao Pang, Wenhao Wang, Hongyi Zhang, Guangxin Tuo, Peng Wu, Zequan Wang, Zijiang Zhu

**Affiliations:** a Department of Clinical Medicine, Gansu University of Traditional Chinese Medicine; b Department of Thoracic Surgery, Gansu Provincial Hospital, Lanzhou, China.

**Keywords:** artificial intelligence, diagnostic accuracy, meta-analysis, protocol, thoracic cancers

## Abstract

**Introduction:**

Thoracic diseases include a variety of common human primary malignant tumors, among which lung cancer and esophageal cancer are among the top 10 in cancer incidence and mortality. Early diagnosis is an important part of cancer treatment, so artificial intelligence (AI) systems have been developed for the accurate and automated detection and diagnosis of thoracic tumors. However, the complicated AI structure and image processing made the diagnosis result of AI-based system unstable. The purpose of this study is to systematically review published evidence to explore the accuracy of AI systems in diagnosing thoracic cancers.

**Methods and analysis:**

We will conduct a systematic review and meta-analysis of the diagnostic accuracy of AI systems for the prediction of thoracic diseases. The primary objective is to assess the diagnostic accuracy of thoracic cancers, including assessing potential biases and calculating combined estimates of sensitivity, specificity, and area under the receiver operating characteristic curve (AUC). The secondary objective is to evaluate the factors associated with different models, classifiers, and radiomics information. We will search databases such as PubMed/MEDLINE, Embase (via OVID), and the Cochrane Library. Two reviewers will independently screen titles and abstracts, perform full article reviews and extract study data. We will report study characteristics and assess methodological quality using the Quality Assessment of Diagnostic Accuracy Studies 2 (QUADAS-2) tool. RevMan 5.3 and Meta-disc 1.4 software will be used for data synthesis. If pooling is appropriate, we will produce summary receiver operating characteristic (SROC) curves, summary operating points (pooled sensitivity and specificity), and 95% confidence intervals around the summary operating points. Methodological subgroup and sensitivity analyses will be performed to explore heterogeneity.

**PROSPERO registration number:**

CRD42019135247

## 1 Introduction

Thoracic diseases include a variety of common human primary malignant tumors, among which lung cancer and esophageal cancer are among the top 10 in cancer incidence and mortality.^[1]^ Though the survival rates of most cancers have improved over the past few decades, lung cancer survival rates have declined, mainly because the cancer is often well advanced, with limited treatment options by the time it is detected.^[2]^ The 5-year survival rate of patients with advanced non-small cell lung cancer is 14% to 15.7%.^[3]^ In contrast, early stages are often curable, and detection during the early stage could drastically improve patient outcomes, minimize overtreatment, and even save lives.^[4–6]^ Although low-dose helical computed tomography (LDCT) can detect more patients with early-stage lung cancer and has few complications, it still requires radiation exposure, has a high false positive rate and may lead to potential overdiagnosis^[7]^; moreover, 96.4% of lung nodules detected by LDCT screening are not cancerous, and there is no definite method to classify whether these nodules are cancerous or benign, which can lead to a greater economic and psychological burden to patients.^[5]^

With the increasing awareness of cancer prevention and the vast amount of data generated every day from parallel streams of screening, doctors need to analyze a large number of medical images. Therefore, it is becoming increasingly important to effectively and accurately identify lesions and optimize and simplify the clinical workflow.^[8,9]^ These new challenges have prompted scientists to develop a reliable early detection method that is not a new imaging technology but a way to address challenges in areas such as cancer detection and monitoring based on medical imaging data.^[10,11]^ It is hoped that this technique can simulate the process of traditional imaging diagnosis, which mainly relies on the qualitative characteristics (texture, intensity shape, etc) of the lesions, the outline of the tumor, and anatomic relationships. The focus of this technique is to master these phenotypic descriptions of lesions, known as “semantic characteristics.”^[12,13]^ Decades ago, scientists began trying to explore a way to use third-party tools to help clinicians extract semantic features. In recent years, with the development of computer equipment and radiomics, artificial intelligence (AI) has made great progress in medical imaging diagnosis of diseases.^[11,14,15]^

AI is a science that applies intelligent machines and systems to imitate the ability of intelligent human activities. The use of AI in the diagnosis of thoracic tumors has come a long way. In 1956, computer-aided detection (CAD)^[16]^ was first proposed to predict the existence of pulmonary nodules. With advances in computer technology, CAD was implemented in the 1980s as a tool for the automatic detection of suspicious pulmonary nodules and their location for radiologists.^[17,18]^ However, due to the difficulty in modeling and low accuracy, CAD has not been well developed. During this period, LeCun designed the first truly convolutional neural network, and the emergence of support vector machine (SVM), random forest, and LSTM also laid the foundation for the subsequent rise of AI. In the last decade, these technologies, together with deep learning, have pushed thoracic cancer diagnosis toward the direction of AI,^[19–21]^ especially for the 3 important functions of disease diagnosis: detection, characterization, and monitoring. AI-based detection tools can be used to reduce oversight, track disease progression, and improve diagnostic accuracy; for example, they can be used to detect and monitor the presence of small nodules in thoracic LDCT and distinguish benign and malignant tumors in thoracic LDCT.^[4,22–24]^ This greatly alleviates the challenges of clinical work.

### 1.1 Background

#### 1.1.1 Artificial intelligence

AI overlaps with computer science and robotics, but it focuses more on empirically processing machine behavior. It is similar to studying animal behavior by combining evolutionary characteristics shaped by physiology, biochemistry, ecology, and the environment.^[25,26]^ AI has shown significant impact and potential value in the following 4 areas related to clinical work: managing population health; as a frontline health worker virtual health assistant; as a patient virtual health assistant; and as physician clinical decision support tools.^[4]^ AI plays important roles in the diagnosis and treatment of tumors, including providing accurate descriptions of changes in disease over time, such as changes in tumor size over time; parallel tracking of multiple lesions; and detecting the association of subtle phenotypic differences within tumors with genotypes^[27,28]^; thus, AI has the potential to improve clinicians’ ability to predict outcomes comprehensively. In simple terms, AI is a classifier that imports the feature semantics required for classification into the classifier and then differentiates the input images through complex image feature analysis to achieve the purpose of diagnosis.^[29–31]^ In addition, AI can also aggregate multiple streams of data into powerful integrated diagnostic systems, such as combining radiographic images with genomic data and pathological grades to thereby complement clinical decision making. It is worth mentioning that deep learning, as a subset of AI, has the ability of automatic learning by mining data to find links that humans cannot detect.^[4,21]^ However, there have been no studies to evaluate which model is better at diagnosing thoracic tumors than traditional machine learning.

#### 1.1.2 Machine learning

Machine learning algorithms can be divided into 3 types: supervised learning, unsupervised learning, and reinforcement learning. Among them, the development of supervised learning has led to the best results.^[32,33]^ Supervised learning obtains a model by training samples and then uses this model for diagnosis. SVM, random forest, and decision tree are the most widely used in the field of thoracic diseases.^[34,35]^ Between 1980 and 2010, machine learning has eclipsed neural networks. Until recent years, there have been many studies using this type of model. However, the development of unsupervised learning in this field has been slow. Although smooth results have been obtained by clustering algorithms (such as the EM algorithm)^[36]^ and data dimensionality reduction (such as Laplace feature mapping),^[37]^ no representative model has been used for thoracic disease diagnosis.

SVM, a representative machine learning method first proposed by Cortes and Vapnik in 1995, has shown many unique advantages in solving the problems of small sample sizes and nonlinearity as well as in high-dimensional pattern recognition and can be generalized and applied to other machine learning problems such as function fitting. SVM is a binary classification model whose basic model is defined as the largest linear classifier in the feature space, which can be transformed into the solution of a convex quadratic programming problem. SVM is a milestone in the diagnosis of thoracic cancers by AI. By implicitly mapping input vectors to high-dimensional space, SVM can handle nonlinear problems well and improve the efficiency of diagnosis.^[38,39]^

#### 1.1.3 Deep learning

The development of deep learning is closely related to neural networks. Convolutional neural networks (CNN) and LSTM have been proposed as early as the 1980s.^[21]^ Unfortunately, neural networks have not been used successfully because of their long time calculation,^[40]^ and they are at a disadvantage compared with machine learning algorithms such as SVM. However, with the increase in data flow and the improvement of accuracy requirements, the ability of machine learning technology to process raw data has begun to show deficiencies. With the advent of high-efficiency GPUs, ReLUs, dropout layer, cross-validation, and other devices and technologies, neural networks have once again become remarkable.^[41–43]^ In 2006, the deep belief network proposed by Hinton opened a new era of deep learning. He proposed to use a neural network to reduce the dimensionality of data, which is called sparse coding or automatic coding.^[44]^

At the same time, the CNN, a particular type of deep, feedforward network, has had many practical successes, even during the period when neural networks were out of favor. However, it was not until 2012, when Alex Net won the ImageNet classification contest, that CNN again attracted attention. CNN is a multilayer structure that is composed of a convolutional layer, nonlinear processing unit, and subsampling layer. The convolutional layer is the core of the CNN and is used to extract the corresponding features of images. Its functions are similar to those of filters (or similar to the retina of the eye). Then, the result obtained by the convolution layer is activated by nonlinear mapping (ReLU). The next pooling layer will compress the input characteristic graph, reduce the spatial dimension of the input data, and simplify the network computing complexity, including maximum pooling and average pooling. To avoid overfitting in this process, the dropout layer regularizes the information, then reduces the dimensions of the output characteristic graph, changes the 2D matrix to 1D, multiplies the weight, and finally passes the output value to the classifier (such as the softmax classifier).^[45,46]^

#### 1.1.4 Radiomics

Radiomics information, which comes from the high-throughput mining of large-scale image features from medical images, enables data to be quantified and applied to the process of AI systems, which significantly affects the accuracy of diagnosis. Radiomics typically consists of 5 components: data selection, medical imaging, feature extraction, exploratory analysis, and modeling. First, we need to determine 3 points: the imaging protocol, the volume of interest (VOI), and a prediction target. To avoid unnecessary confounding variability, a standard imaging protocol should be selected. Then, there is image segmentation, where VOIs are obtained manually or (semi-) automatically. This stage usually determines which voxels in the image are analyzed, so the process is prone to introducing biases in evaluating derived radiological features, which may also be a factor in the differences between different diagnostic models. Next comes the core of radiomics, which is the high-throughput extraction of quantitative image features to characterize VOIs. The feature eigenvalues depend on factors that can include image preprocessing (e.g., filtering or intensity) and reconstruction (e.g., iterative reconstruction). In addition, it is also influenced by tag naming, algorithms, and software implementation of the feature extraction algorithm. Next, the features from the previous step are combined with the prediction target to create a single dataset. Finally, the previously generated single dataset is combined with the AI model to form the “semantic feature” set, and the images that are later used for classification are also quantified according to the above steps.^[15,47,48]^

### 1.2 Objective

However, the complicated AI structure and image processing made the diagnosis result of AI-based system unstable. Therefore, to establish a more comprehensive and extensive summary of the AI diagnostic models for thoracic cancers, we will conduct a systematic review and meta-analysis. Our main objective is to assess the accuracy of the diagnosis of thoracic diseases, including assessing potential biases and calculating combined estimates of sensitivity, specificity, and area under the receiver operating characteristic curve (AUC). The second objective is to evaluate factors related to diagnostic accuracy and different models (machine learning vs deep learning), classifiers (including CNN, SVM, random forests, LSTM, etc), and radiomics information (image segmentation methods, feature extraction methods).

## 2 Methods

### 2.1 Search strategy

This review adopts recommendations based on the conduct and reporting of systematic reviews and meta-analyses outlined by the Preferred Reporting Items for Systematic Reviews and Meta-analyses statement. We will search the following databases for relevant studies: PubMed/MEDLINE, Embase (via OVID), and the Cochrane Library, for reports on the diagnostic accuracy of AI in thoracic diseases published between 1946 and December 2019. There will be no language restrictions. We developed a search strategy using a combination of keywords and medical subject headings (MeSH)/EMTREE terms, and the following expressions will be used:(Endoscope OR gastroscope OR “Spiral Cone-Beam Computed Tomography” OR CT OR CAT) AND (((nodule OR cancer OR tumor) AND (lung OR pulmonary OR thoracic)) OR ((“squamous cell carcinoma” OR carcinoma OR cancer OR neoplasm OR “adenosquamous carcinoma” OR “basosquamous carcinoma” OR Barrett's) AND (esophageal) OR esophagus))) AND (Computer aided OR Machine learning OR Deep learning OR Algorithms OR Artificial Intelligence).

### 2.2 Eligibility criteria and exclusion criteria

#### 2.2.1 Eligibility criteria

All the articles will be assessed independently by 2 researchers. The inclusion criteria are as follows: assessment of the diagnostic accuracy of AI for diagnosing thoracic cancer (including early lung cancer and early esophageal cancer); medical imaging data including CT images and white light endoscopic images; and inclusion of sensitivity, specificity, or sufficient information to construct the 2 × 2 tables (outcome).

#### 2.2.2 Exclusion criteria

We will exclude studies from which we cannot obtain or calculate the data from the text, appendices, or after contacting the main authors; moreover, duplicates or subcohorts of already published cohorts, reviews, case reports, conference abstracts, animal experiments, and meta-analyses will be excluded from the study.

### 2.3 Study selection and data extraction

We will use EndNote software (Thomson Reuters, Toronto, Ontario, Canada) to store and remove duplicate references. The articles will be screened independently by the 2 authors (GJ, GXT) based on the titles and abstracts, and those not meeting the criteria will be eliminated. Disagreements will be solved by discussion and submission to the third author (WHW) if necessary. After this initial phase, the full texts of all remaining articles will be independently reviewed by the 2 authors (GJ, GXT) for inclusion or exclusion in the final study. Conflicts will be resolved in the same way as when they were initially screened.

Two authors (YP, PW) will extract the study characteristics from each included study, and the extraction of data from the selected articles will be independently performed by 2 investigators. Disagreements will be resolved through discussion and consensus or by consulting a third member (YY) of the review team. The data to be collected will include the following: author, year of publication, number needed to treat, race, model, algorithm, image source, diagnostic gold standard, sensitivity (SEN), specificity (SPE), true positive (TP) rate, false positive (FP) rate, false negative (FN) rate, true negative (TN) rate, nodule segmentation, feature extraction and selection, nodule classification, and AI for diagnosing thoracic disease.

### 2.4 Quality evaluation

Two reviewers (HYZ, ZQW) will appraise all the included studies by using a checklist based on the Quality Assessment of Diagnostic Accuracy Studies 2 (QUADAS-2) guidelines. The QUADAS-2 includes 4 parts regarding patient selection, index test, reference standard, and flow and timing risk of bias. The risk of bias is classified as “low,” “high,” or “unclear.”^[49]^

### 2.5 Data analysis

We will first extract the 2 × 2 contingency table (TP, FP, TN, FN). Some primary studies do not directly provide all the data in a 2 × 2 table. We will calculate the missing data based on the existing data in the paper, such as SEN, SPE, and the number of cases, through the calculator in Review Manager 5.3. For the meta-analysis, the pooled SEN and SPE with 95% confidence intervals for diagnosis by AI will be calculated. The descriptive forest plot and summary receiver operating characteristic (SROC) curves will be derived by Meta-disc 1.4. We will plot the 95% CI and prediction regions around the averaged accuracy estimates in the SROC space, and the AUC will be calculated. SROC curves are defined by sensitivity (*y*-axis) and specificity (*x*-axis), and AUC is the final comparison indicator. The criteria for AUC classification are 0.90 to 1 (excellence), 0.80 to 0.90 (good), 0.70 to 0.80 (fair), 0.60 to 0.70 (poor), and 0.50 to 0.60 (failure).^[50]^

### 2.6 Assessment of heterogeneity

Initially, we will visually inspect the forest plots of each study's sensitivities and specificities as well as SROC curves related to the individual study results to examine heterogeneity. Statistical heterogeneity will be evaluated informally from the forest plots of the study estimates and more formally using the *I*^2^ statistic (*I*^2^ > 50% = significant heterogeneity). In addition, different diagnostic thresholds of the included studies may lead to heterogeneity; we will use the Spearman correlation coefficients to test whether there is a threshold effect. When there is a threshold effect, sensitivity and specificity will be negatively correlated, and the results will present a “shoulder-arm” point distribution on the SROC curve.

### 2.7 Publication policy

Publication bias will be explored with Begg and Egger funnel plots.

## 3 Discussion

In recent years, with the emergence of deep learning technology, many AI models have been used for the diagnosis of thoracic diseases, but the accuracy of these models varies greatly. Although there is evidence that AI can improve the accuracy of thoracic cancer diagnosis, the evidence is limited, and there has been no systematic evaluation. We will use appropriate methods and quality assessment tools to systematically evaluate the models used to diagnose thoracic diseases and provide evidence for clinical practice. When establishing a diagnostic strategy that can accurately reflect the current evidence, greater scientific rigor is needed. We will also conduct subgroup analysis based on the model used, the type of classifier, and radiomics information.

One major limitation is that the proportion of malignant tumors in the original study sample is much higher than that in the normal population, which may lead to overfitting and more optimistic results. In addition, the original studies are mainly retrospective, and the quality of the original studies will affect the quality of the systematic evaluation.

## Author contributions

**Conceptualization:** Zijiang Zhu.

**Investigation:** Yi Yang, Gang Jin, Yao Pang, Hongyi Zhang.

**Methodology:** Yi Yang, Gang Jin.

**Project administration:** Yi Yang, Gang Jin, Wenhao Wang, Guangxin Tuo, Peng Wu, Zequan Wang.

**Supervision:** Zijiang Zhu, Yi Yang.

**Writing – original draft:** Yi Yang, Gang Jin.

**Writing – review & editing:** Yi Yang, Gang Jin, Yao Pang, Hongyi Zhang.

Yi Yang orcid: 0000-0001-5801-0170.
